# Antioxidant Bioaccessibility of Cooked Gluten-Free Pasta Enriched with Tomato Pomace or Linseed Meal

**DOI:** 10.3390/foods13223700

**Published:** 2024-11-20

**Authors:** Lorenzo Estivi, Gabriella Pasini, Amel Betrouche, Vanja Traviĉić, Elena Becciu, Andrea Brandolini, Alyssa Hidalgo

**Affiliations:** 1 Department of Food, Environmental and Nutritional Sciences (DeFENS), Università degli Studi di Milano, Via Celoria 2, 20133 Milan, Italy; lorenzo.estivi@unimi.it (L.E.); elebeb97@gmail.com (E.B.); 2 Department of Agronomy, Food, Natural Resources, Animals and Environment (DAFNAE), Università degli Studi di Padova, Viale Dell’Università, 16, 35020 Legnaro, Italy; gabriella.pasini@unipd.it; 3 Laboratoire de Génie Agro-Alimentaire, Institut de la Nutrition, de l’Alimentation et des Technologies Agroalimentaires (GéniAAl-INATAA), Université des Frères Mentouri Constantine 1, 325 Route de Ain El Bey, Constantine 25017, Algeria; abetrouche@live.fr; 4 Faculty of Technology Novi Sad, University of Novi Sad, Bulevar Cara Lazara 1, 21000 Novi Sad, Serbia; vanjaseregelj@tf.uns.ac.rs; 5 Research Centre for Animal Production and Aquaculture (CREA-ZA), Consiglio per la Ricerca in Agricoltura e l’Analisi dell’Economia Agraria, Via Piacenza 29, 26900 Lodi, Italy; andrea.brandolini@crea.gov.it

**Keywords:** antioxidant capacity, bioactive compounds, carotenoids, flavonoids, gluten-free pasta, polyphenols, tocols

## Abstract

Gluten-free products lack bioactive compounds, while vegetable wastes from food manufacturing are still rich in nutrients. This study compared the antioxidants of gluten-free pastas enriched with vegetable by-products: the control formulation (66.7% rice and 33.3% fava bean flours) was enriched with 10% or 15% of tomato waste (TO) or defatted linseed cake (LI). Carotenoids, tocols, phenolics, and antioxidant capacity (ABTS and FRAP) were determined in the cooked pasta as well as in the soluble and insoluble fractions after *in vitro* gastro-intestinal digestion. The cooked enriched pastas showed higher levels of carotenoids (1.36–1.53 vs. 1.02 mg/kg DM), except for the LI-added samples, tocols (8.83–21.70 vs. 7.01 mg/kg DM), free polyphenols (218.1–258.6 vs. 200.9 mg/kg DM), bound polyphenols (132.7–177.6 vs. 101.9 mg/kg DM), and antioxidant capacity. Cooking augmented the carotenoids and free polyphenols in the enriched pastas, tocols in LI pastas and bound flavonoids in TO pastas. After digestion, the recoveries for soluble and insoluble fractions were 53% and 35% for carotenoids, 52% and 43% for tocols, 109% for free phenolic acids, 97% for free flavonoids, 93% for bound phenolic acids, and 100% for bound flavonoids. Bioaccessibility was the highest for free phenolic compounds, whereas carotenoids and tocols were partially available.

## 1. Introduction

The consumption of gluten-free foods has significantly increased among people with eating problems as well as among other groups of consumers [1]. To mimic the unique viscoelastic properties of gluten, in gluten-free pasta the protein network is replaced by a retrograded starch matrix obtained from cereal flours (e.g., rice, corn, sorghum), pseudocereals, or legumes, to which structuring additives such as gums, hydrocolloids, proteins, and emulsifiers are added [2]. Compared to traditional pasta, gluten-free pasta shows an increase in high-glycemic index carbohydrates, fats, and sodium, while fibre, B vitamins, and minerals are reduced [3]. This suggests that consumers of gluten-free pasta may be at greater risk of developing obesity, diabetes, and cardiovascular diseases [4]. Food manufacturing generates large amounts of waste, whose disposal is often expensive and represents a threat for the environment [5]. Vegetable by-products still contain numerous nutrients, in particular fibres, proteins, antioxidant compounds, vitamins, and minerals that can be added to food products to improve their nutritional value [5,6,7]. Hence, reusing these by-products would offer an opportunity to optimise the nutritional profile of gluten-free foods and at the same time would reduce waste volume, thus improving the sustainability of the agri-food system in the optic of a circular economy [8].

A study on the composition of five types of gluten-free pasta, one obtained from rice and fava bean flour, two enriched with 10 or 15% tomato waste, and two enriched with 10 or 15% linseed cake showed that the enriched pastas had better antioxidant compound profiles and higher fibre content [9]. Similarly, the optimised addition of cold-pressed pumpkin and okra seed by-products in gluten-free (GF) noodles had a positive impact on protein (43%) and dietary fibre (40%), as well as on sensory attributes [10], while the supplementation of chestnut flour increased the content of free phenolic acids, total polyphenols, and antioxidant capacity [11]. GF pasta enriched with chickpea hulls had a higher level of bioactive compounds and antioxidant activity than the control [12]. Pasta supplementation with food by-products, often rich in bioactive and antioxidant compounds, has emerged as a worthy strategy to improve its nutritional composition [13]. However, only a limited number of studies characterised the changes of these compounds during simulated gastro-intestinal digestion. In particular, to the best of our knowledge, most of these studies tested bioaccessibility using a spectrophotometric approach (i.e., Folin–Ciocalteu), rather than HPLC methods.

Therefore, the aim of this research was to investigate the evolution of the main antioxidants in gluten-free pastas enriched with vegetable by-products (tomato waste or cold-pressed linseed cake) after cooking and after *in vitro* digestion. To this end, the contents of tocopherols, tocotrienols, carotenoids, and phenolic compounds, as well as antioxidant capacity, were assessed in the cooked pasta and in the soluble and insoluble fractions obtained after its *in vitro* gastro-intestinal digestion.

## 2. Materials and Methods

### 2.1. Materials

The raw materials for the preparation of the control pasta were rice flour (<200 µm) obtained from Bio Aglut Company (Constantine, Algeria) and fava bean flour (<200 µm) prepared by grinding seeds purchased from Al-Amir Company (Housh Essa, Egypt). The by-products used for the manufacturing of the enriched pastas were tomato waste powder (TO), obtained by drying at 45 °C for 10 h and then grinding a tomato pomace acquired from Zimba Canning Company (Guelma, Algeria), and linseed meal (LI), the by-product of mechanical oil extraction, supplied by Health Embassy LTD (Cheltenham, UK).

### 2.2. Sample Preparation

#### 2.2.1. Pasta Making

The five different pastas were prepared as described by Betrouche et al. [9]. Briefly, the control gluten-free pastas were made from 66.7% rice flour and 33.3% fava bean flour, while the enriched pastas were prepared by replacing 10% or 15% of the basic formulation with tomato waste (TP) or linseed meal. The pasta samples were dried in an experimental cell (Braibanti, Milan, Italy) using a long drying cycle (17 h) at low temperature (max 60 °C) and relative humidity of 75%. All pasta samples were stored at room temperature (20 °C) until cooking.

#### 2.2.2. Pasta Cooking

Before antioxidant analysis and digestion, 15 g of pasta was boiled for 8 min in 180 mL of deionised water on a glass–ceramic electric plate, using beakers covered with aluminium foil to prevent excessive evaporation. The optimal cooking time was determined for all the samples, starting from 6 min with 1 min step increases up to 10 min, by visual evaluation, i.e., cutting the macaroni and checking the progressive disappearance of the non-hydrated area in the centre of the product. After cooking, the samples (Figure S1) were frozen and stored at −20 °C. Three different batches of pasta were cooked, underwent digestion, and were analysed.

#### 2.2.3. Cooked Pasta Milling

The frozen samples were ground at 18,000 rpm for 20 s with a Waring Heavy Duty Blender (Waring Commercial, Torrington, CT, USA) using a blade suitable for dusty materials and screened with an 18-mesh steel sieve, to exclude particles larger than 1 mm. The samples were then stored at −20 °C in sealed vacuum bags until analysis.

#### 2.2.4. *In Vitro* Digestion

The *in vitro* digestion was carried out on the cooked pasta following Minekus et al. [14]. Briefly, 2.5 g of sample was mixed with 2.5 mL simulated salivary solution (SSF) at pH 7.0 and α-amylase (75 U/mL SSF) for 2 min, to reproduce the oral phase of digestion. The bolus obtained was subsequently mixed with 5 mL simulated gastric juice (SGF) containing porcine pepsin (2000 U/mL SGF). Gastric digestion was performed at 37 °C for 2 hat pH 3.0 (adjusted with 1 N HCl). To obtain the intestinal digest, 10 mL of simulated duodenal secretions (SDF) and bile salts (10 mM, Sigma-Aldrich, St. Louis, MO, USA) were added to the gastric digests. The enzymes used for intestinal digestion were porcine trypsin (200 U/mL SDF), bovine chymotrypsin (50 U/mL SDF), pancreatic amylase (200 U/mL SDF), porcine intestinal lipase (4000 U/mL SDF), and co-lipase (maximum co-lipase:lipase ratio = 1:2). This step was carried out at 37 °C for 2 h at pH 7.0 (adjusted with 1 N NaOH) and stopped by adding the protease inhibitor 4-(2-aminoethyl) benzenesulfonyl fluoride (Roche, Mannheim, Germany) at a 1 mM total concentration. The supernatants were separated from sediments by centrifugation; both fractions were then lyophilised and kept at −20 °C. A “blank”, useful for identifying any disturbances and for calculating the results, was obtained by running both digestive phases without the addition of the samples.

For each compound, the proportion released from the food matrix or control solution into the digestive fluids and the proportion insoluble during digestion were computed as:

Soluble (%) = (Supernatant/Digested) × 100(1)

Insoluble (%) = (Pellet/Digested) × 100(2)

where Supernatant is the compound quantity (mg) in the supernatant at the end of the digestion phase, Pellet is the compound quantity (mg) in the pellet at the end of the digestion phase, and Digested is the compound quantity (mg) submitted to digestion.

The total recovery of each antioxidant group was computed as:

Total recovery = Soluble (%) + Insoluble (%)(3)

### 2.3. Analyses

#### 2.3.1. Carotenoid and Tocol Quantification

The moisture of the cooked pastas was determined gravimetrically [15] to report the results as dry matter basis (DM). Carotenoid and tocopherol extracts from cooked pastas and digested pastas were analysed as previously described [16]. Briefly, 2 g cooked pasta or 1.5 g lyophilised digested samples was exactly weighted in a screw-capped tube and saponified under nitrogen for 45 min at 70 °C, with the addition of 5 mL ethanolic pyrogallol (60 g/L) as antioxidant, 2 mL ethanol (95%), 2 mL sodium chloride (10 g/L), and 2 mL potassium hydroxide (600 g/L). Afterwards, they were cooled in an ice bath, and 15 mL sodium chloride (10 g/L) was added. The suspension was then extracted twice with 15 mL hexane:ethyl acetate (9:1 *v/v*). The organic layer was collected and evaporated under vacuum, followed by nitrogen drying; the residue was dissolved in 2 mL hexane:isopropyl alcohol (99:1 *v/v*) and filtered through a 0.22 mm PTFE membrane.

Carotenoid and tocol quantification was carried out by normal-phase chromatography [16]. The operating conditions for carotenoid detection were injector Rheodyne 7125 (VWR, Hitachi, Tokyo, Japan) mounting a 50 μL loop; mobile phase, hexane:2-propanol 95:5 (*v/v*); isocratic flow rate, 1.5 mL/min; pump L-2130 Elite LaChrom (Hitachi, Tokyo, Japan); Alltima Silica column 250 × 4.6 mm, 5 μm and Alltima Silica guard column 7.5 × 4.6 mm, 5 μm (Alltech Associates Inc., Deerfield, MA, USA). The carotenoids were detected at 445 nm by a 2996 PhotoDiode Array Detector (Waters Chromatography Division, Millipore, Milford, MA, USA) set in the range of 200–650 nm and connected to the Empower 2 software (Waters).

The operating conditions for tocol quantification were injector Rheodyne 7125 (VWR, Hitachi, Tokyo, Japan) mounting a 50 μL loop; mobile phase, hexane:ethyl acetate:acetic acid 97.3:1.8:0.9 (*v/v/v*); isocratic flow rate, 1.6 mL/min; pump L-2130 Elite LaChrom (Hitachi, Tokyo, Japan); Alltima Silica column, 250 × 4.6 mm, 5 μm and Alltima Silica guard column 7.5 × 4.6 mm, 5 μm (Alltech Associates Inc., Deerfield, MA, USA); and fluorimetric detector 821-FP Intelligent Spectrofluorometer (Jasco, Tokyo, Japan) set at excitation–emission wavelengths of 290 nm and 330 nm, respectively, and connected to the Empower 2 software through the e-SAT/IN module (Waters).

All the compounds were quantified by the external standard method and the results are expressed as mg/kg DM.

#### 2.3.2. Phenol Quantification

Soluble free and insoluble bound phenolics, extracted from 1 g cooked pastas and 1.5 g from digested pastas, were extracted and analysed as previously outlined [16] using an HPLC system with an Adamas^®^ C18-AQ 5 μm 4.6 mm × 250 mm column and a C18 5 μm 4.6 mm × 10 mm precolumn (Sepachrom srl, Rho, Italy), thermostated at 30 °C as well as an L-2130 pump, L-2300 column oven, and L2450 Diode Array Detector Elite LaChrom (Hitachi, Tokyo, Japan). The mobile phase consisted of a gradient of formic acid 1% *v/v* in water and acetonitrile. The identity of the compound was confirmed by the congruence of retention times and UV/VIS spectra with those of pure authentic standards. The chromatogram revealed the presence of the amino acid tryptophan. Since the method was not specifically designed to measure this compound, the determination must be considered semi-quantitative. The unidentified peaks were quantified using the calibration curve of the compound with a similar absorption spectrum and named as “phenolic derivative”. All the compounds were quantified by the external standard method, and the results are expressed as mg/kg DM.

#### 2.3.3. Antioxidant Capacity Tests

The antioxidant capacity of the same extracts analysed by HPLC for phenol quantification was determined using the 2,2′-azino-bis(3-ethylbenzothiazoline-6-sulfonic acid) (ABTS) [17] and Ferric Reducing Antioxidant Power (FRAP) [18] methods. The ABTS test was performed by adding 150 µL of extract to 3.0 mL of a radical solution, consisting of 10 mL of a 7 mM aqueous ABTS solution and 170 µL of a 140 mM aqueous potassium persulfate solution, with an absorbance value of 0.70 ± 0.02. After 6 min of reaction at 30 °C in a thermostated bath (EN.CO. Srl, Spinea, Italy), the absorbance was measured at 734 nm using plastic cuvettes with a JASCO V-650 Spectrophotometer (Tokyo, Japan). For the FRAP test, exactly 3.0 mL of the FRAP reagent was added to 150 µL of the extract sample, and the absorbance was measured at 593 nm after 60 min of reaction at 37 °C in a thermostated bath. The results are expressed as mmol Trolox equivalent (TE)/kg DM.

### 2.4. Statistical Analysis

All the chemical analyses were performed on three independent samples. To evaluate the effect of the different pasta formulations, the data were processed by one-way analysis of variance (ANOVA), considering the type of mixture as a factor. Before the ANOVAs, the data normal distribution was verified. When significant differences were found (*p* < 0.05), Fisher’s least significant difference (LSD) at 95% significance was computed. All analyses were performed using the statistical program STATGRAPHICS^®^ Centurion 18 (Statpoint Technologies, Inc., Warrenton, VA, USA). The means and standard deviations were calculated using the Excel program (Microsoft^®^, Redmond, WA, USA).

## 3. Results

### 3.1. Carotenoids

Limited quantities of lycopene + β-carotene (more abundant in the tomato waste enriched samples), β-cryptoxanthin, lutein, and zeaxanthin were identified in both the cooked and the digested pasta samples (Table 1). All the enriched samples showed higher carotenoid contents (*p* < 0.05) than the control. In particular, the pastas with TO and with 15% LI contained between 30% and 50% more carotenoids compared to the control, depending on the percentage of enrichment.

By comparing our results to those of the very same uncooked samples reported by Betrouche et al. [9], a decrease in total carotenoid content (from 1.17 to 1.02 mg/kg DM; *p* < 0.05) was observed in the control. A slight increase (from 0.91–1.40 to 1.35–1.53 mg/kg DM; *p* < 0.05) was instead observed in the enriched pastas (except for 10% LI), possibly due to improved extraction resulting from heat-induced loss of cellular compartmentalisation and/or rupture of their bonds with other substrates, as previously reported [19,20]. A limited carotenoids decrease after cooking in durum wheat pasta and/or pasta enriched with encapsulated carotenoids was already described [21].

**Figure 1:**
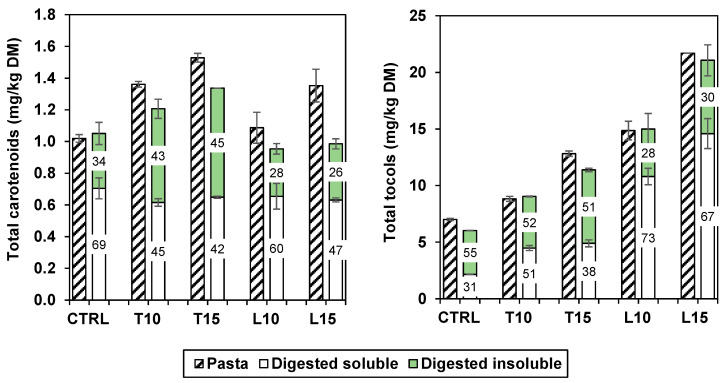
Carotenoid and tocol content of cooked pasta (striped left column) and digested pasta (plain right column; soluble fraction in white, insoluble fraction in green). The numbers indicate the recovery percentages after digestion.

The carotenoids of the *in vitro* digested pasta were recovered from two different phases, soluble and insoluble. The results, summarised in Figure 1 and presented in detail in Table 1, demonstrate that the *in vitro* digestion slightly reduced (*p* < 0.05) carotenoid content, especially in the enriched pasta. The total carotenoid recovery in the soluble fraction of the enriched pastas varied between 42% and 60% (average: 53%) and was lower than that of the control pasta (69%). Higher recovery values (between 65% and 83%) were reported in einkorn wheat biscuits enriched with pseudocereals and in their control [22]. The total carotenoid recovery (soluble fraction + insoluble fraction) was around 88%. Werner and Böhm [23] recovered 96–98% of carotenoids after digestion of durum wheat pasta, of which 27–40% was bioaccessible. Such a high recovery might be attributed to the presence of an antioxidant, pyrogallol, that we did not employ during digestion. The same authors demonstrated that the amount of bile salts during digestion is crucial in the solubilisation of carotenoids and tocols: in fact, they found that increasing bile salts 5-fold augmented the recovery up to 57% in egg pastas and up to 63–78% in wheat pasta. Oduro-Obeng et al. [20] reported bioaccessibility values of all-*trans* lutein ranging from 101% to 157% in digested samples of pasta made with whole durum wheat or refined semolina. These values, exceeding 100%, are attributable to the use of mild extraction conditions (i.e., liquid extraction in ethanol/water solution), thus effectively comparing the carotenoids in the digested supernatant with those already soluble in the undigested sample, rather than with total carotenoids as in the present study.

The fate of carotenoids during *in vitro* digestion of pumpkin pulp was studied by Lyu et al. [24]; they found that carotenoids gradually increase in the supernatant, probably because of the mechanical action that facilitates their release from the chloroplasts; however, the solubilised carotenoids only minimally compensated the decrease in the sediment, indicating a degradation, probably due to their instability in regard to the simulated gastric acidity (pH 2.5) [23].

### 3.2. Tocols

Tocol quantification identified different homologues in cooked pasta (Table 2). The control, prepared exclusively from rice and fava bean flours, contained only γ-tocopherol and γ-tocotrienol, while the pasta with tomato waste also had α-tocopherol, and that with linseed cake β-tocotrienol. Overall, the enriched pasta samples had a tocol content significantly higher (*p* < 0.05) than the control, between 26% and 83% in those with TO and between 112% and 210% in those with LI. As for the carotenoids, a higher by-product concentration led to a higher tocol content. Compared to the same raw pasta [9], no differences were observed in the control and in cooked pasta with tomato waste, while an increase (from 13.15–17.85 to 14.86–21.70 mg/kg DM; *p* < 0.05) was evident in the linseed cake-enriched samples. Conversely, a limited decrease in these compounds after cooking durum wheat pasta enriched with encapsulated tocols was related [21].

Table 2 shows the results of the digested pasta samples. In addition to those already present in cooked pasta, two other tocols were identified in small amounts in the soluble fraction, i.e., β-tocopherol (in all enriched samples) and δ-tocopherol (only in the control). The β-tocopherol was also observed in the digested insoluble fraction of LI-enriched pastas. Figure 1 depicts the total tocol contents in cooked pasta and in the extracts after digestion. On average, the recovery of the tocols from the soluble and insoluble fractions was 52% and 43%, respectively, for a total recovery of about 95%. The soluble fraction percentages were superior in the enriched pasta compared to the control pasta (38–73% vs. 31%), with markedly higher levels in the pasta with LI.

A loss in total tocols was noticed after digestion, as previously observed in the case of biscuit digestion [22]. Likewise, Hossain and Jayadeep [25] perceived that tocopherols and tocotrienols in maize samples underwent a post-*in vitro* digestion reduction compared to the cooked sample. Slightly lower shares of tocols in the soluble fraction (32–45%) were previously determined in digested wheat pasta, but, as mentioned above for carotenoids, far higher values (up to 75%) were obtained when an additional amount of bile salts was used [23]. Conversely, Conti et al. [26] recorded the halving of α-tocopherol after cooking of olive leaf-enriched pasta and its disappearance after digestion.

### 3.3. Free Soluble Polyphenols

Numerous free phenolic compounds were identified in the cooked pasta (Table 3). Protocatechuic, syringic, *p*-coumaric, and ferulic phenolic acids were found in all samples, while *p*-hydroxybenzoic acid was specific to the control; likewise, the flavonoids catechin, epicatechin, quercetin derivative, and apigenin appeared in all samples, but rutin, quercetin, and naringenin were detected only in the pasta with tomato pomace. A UV-absorbing amino acid, tryptophan, was also detected in low concentrations (4.8–21.5 mg/kg DM) in the HPLC chromatogram. The most common compound, the flavonoid catechin, accounted for 52% of all free polyphenols (range: 42–62%), followed by protocatechuic acid (19%). Weighed against the control, the enrichment with TO increased free phenolic acids between 9% and 19% and flavonoids between 23% and 34%, while enrichment with LI showed smaller increases (3% and 11–15%, respectively).

Comparing these data to those of raw dry pastas [9], a significant increase (*p* < 0.05) after cooking in total phenolic acids (on average from 26.0 to 72.5 mg/kg DM) and total flavonoids (from 50.6 to 155.2 mg/kg DM) was observed; overall, the free phenolics increase was from 76.6 to 227.6 mg/kg DM. In fact, cooking alters the food matrix, enhancing the release of soluble phenolics, whose amount reflects a balance between increased losses and improved extractability, and thus, bioaccessibility [13].

After digestion, some compounds (*p*-hydroxybenzoic acid, rutin, quercetin, quercetin derivative, and apigenin) disappeared. Catechin and protocatechuic acid showed concentrations similar to those of the cooked pasta. Meanwhile, tryptophan increased manyfold (*p* < 0.05), ranging from 227.8 mg/kg DM in the control to 403.5 mg/kg DM in pasta with 15% LI, because of protein hydrolysis.

**Figure 2:**
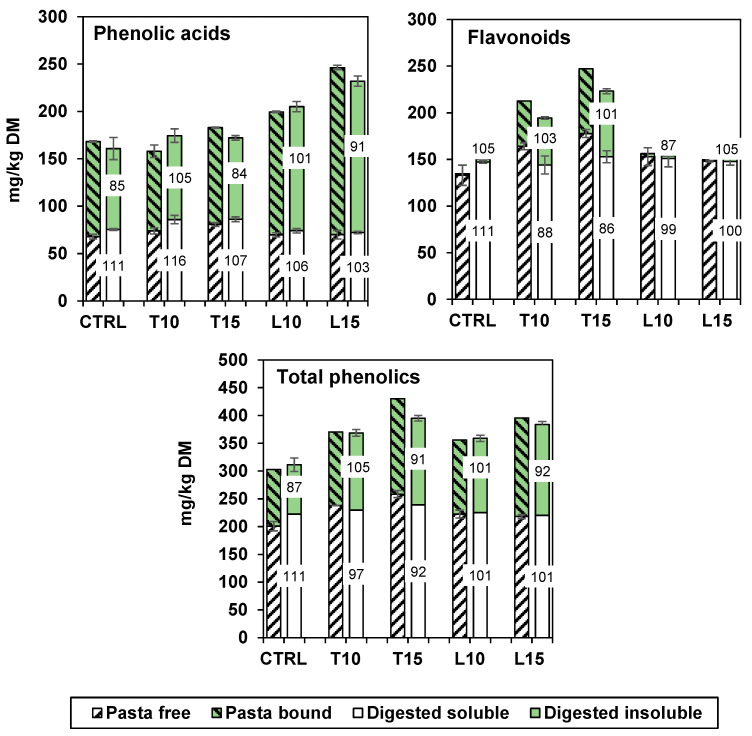
Total soluble (white) and insoluble (green) polyphenol content of cooked pasta (left striped column) and digested pasta (plain right column). The numbers indicate the recovery percentage after digestion for both free (soluble) and bound (insoluble) fractions.

Figure 2 summarises the total free and bound phenolic acids, flavonoids, and total phenolic contents of the cooked pasta and of the extracts after *in vitro* digestion. A total recovery after digestion (103–116%) was observed for free phenolic acids. Similar results were obtained by Liyana-Pathirana and Shahidi [27], who saw phenolic acids increase after gastric digestion, perhaps as a consequence of acidity. Gallic and protocatechuic acids surging after gastrointestinal digestion of quinoa-enriched bread have been observed [28], and the same trend was documented in whole wheat bread, biscuits, and cookies in relation to *p*-hydroxybenzoic, vanillic, ferulic, and sinapic acids [29]. In pasta samples enriched with persimmon flour, total free phenolic acid decreased by 36% after cooking, on average, and by a further 25% after digestion, with a positive contribution coming from protocatechuic and ferulic acids, which increased by 80% or 52%, while flavonoids decreased or disappeared, as also observed in the present study [30].

The variations in phenolic acids after digestion seem to be highly matrix-dependent, since an increase was documented in sorghum samples, while a reduction was seen in sorghum-enriched pastas [31]. Similarly, a 2.3-fold increase in phenolic acids was reported during the digestion of acorn flour, with the highest contribution coming from protocatechuic, *p*-hydroxybenzoic, and vanillic acids, but no changes or minor reductions were documented in acorn-enriched semolina pasta [32].

For the phenolic acids, the soluble fraction accounted for about 50% in the control and TO pastas, but only for about one-third in the LI pastas. In the TO-enriched pasta, where the soluble flavonoids were the vast majority (84–85%) of total flavonoids, a slight decrease in the digestates was noticed; similarly, another flavonoid, rutin, was previously reported to decrease after digestion [28]. An increase in total polyphenol content (TPC) was reported in ditalini pasta “from the post-gastric phase onwards” [33]. On the other hand, Podio et al. [34] observed a certain release of bound polyphenols during cooking but stated that only a small fraction of free polyphenolic compounds was absorbed, i.e., bioavailable, in the small intestine. Compared to cooked pasta before digestion, an increase in TPC concentration both in the control and in red grape marc-enriched pasta, after *in vitro* digestion, was described [27]. After digestion, TPCs were released from a berry-enriched pasta matrix reaching a 2.1- to 3.0-fold increase [35], as well as from pasta enriched with sprouted pseudocereals, whose digestates showed also higher antioxidant capacity [36]. It has been suggested that such increases may be due to pancreatin disrupting the bonds between macromolecules (proteins and fibre) and flavonoids, releasing them from the bound fraction [37]. However, as acknowledged by several authors [29,37,38,39], digestion releases reducing sugars and amino acids, particularly tryptophan, tyrosine, and cysteine, that are detected by the Folin–Ciocalteu reagent, leading to mistakenly inferring a release of phenolic compounds when only TPC determination is performed. In fact, in the present study, while phenolic compounds remained stable overall, tryptophan increased substantially.

### 3.4. Bound Insoluble Polyphenols

The bound insoluble polyphenols found in the cooked pasta (Table 4) were the ferulic (on average, 82% of the bound form), protocatechuic, *p*-hydroxybenzoic, caffeic, *p*-coumaric, sinapic, and cinnamic-derivative phenolic acids, as well as the flavonoids quercetin and naringenin, particularly abundant in the cooked pasta enriched with TO. The bound phenolic acids were more abundant (*p* < 0.05) in the cooked pasta with LI (34–81%) than in TO or the control, while the bound flavonoids, almost absent in the control pasta and in the LI-enriched pasta, increased significantly (*p* < 0.05) after TO addition. Compared to the data of uncooked pasta [9], after cooking the phenolic acid content did not change significantly, and the total flavonoids increased only in the TO-enriched pastas (from 39.6 to 64.7 mg/kg DM).

The digested pasta contained the same polyphenols of the cooked pasta, with the addition of small amounts of epicatechin and naringenin derivative. Ferulic acid was again the main phenolic acid, while naringenin and quercetin were the most relevant flavonoids in the TO-enriched pastas, but they were absent in the other samples. Protocatechuic acid was no longer detectable in the digestates of control pasta and TO-enriched pastas. Overall, the recovery after digestion of total phenolics varied between 87% and 105% for all samples (Figure 2). Previously, losses in bound polyphenols (averaging −32%) were reported after digestion of semolina pasta with added persimmon flour, primarily due to reductions in gallic and ferulic acids [30].

The sum of free and bound polyphenols in the digestates was like that in the cooked pasta samples. The total recovery of phenolic acids and flavonoids was 98%, a percentage similar to that previously reported for biscuits [22]. Bioaccessibility therefore appeared to be the highest for the free phenolic compounds, while the lipophilic antioxidants (carotenoids and tocols) were only partially available for absorption. However, even the insoluble fraction can perform an antioxidant function in the intraluminal side; in particular, the bound phenolic compounds can be hydrolysed by the resident microbiota, helping to protect enterocytes and participating in the reduction of colorectal cancer incidence [40,41].

### 3.5. Antioxidant Capacity

**Figure 3:**
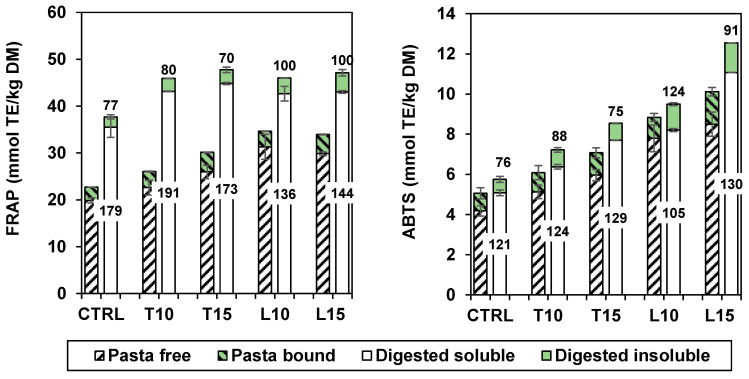
Antioxidant capacity (FRAP and ABTS methods) of the soluble (white) and insoluble (green) fractions of cooked pasta (left striped column) and digested pasta (right plain column). The numbers indicate recovery percentage after digestion for both soluble and insoluble fractions.

The antioxidant capacity of cooked pasta and *in vitro* digested pasta, determined with the FRAP and ABTS assays, is shown in Figure 3. It is evident that the antioxidant capacity was mainly due to the soluble compounds. Furthermore, the digestion increased this capacity significantly (*p* < 0.05) in relation to the augment of tryptophan (Table 3) and, likely, of bioactive peptides [29] in the digestates. The increase in antioxidant capacity was proportionally greater for FRAP, but both methods showed a significant increase in antioxidant activity due to enrichments. FRAP better highlighted the effect of *in vitro* digestion, but the pasta with 15% LI had always the greatest antioxidant capacity. The same behaviour between the two assays was described by Camelo-Mendez et al. [42] for the digestion of gluten-free spaghetti enriched with plantain, chickpea, or blue maize.

Several authors observed an increase in antioxidant capacity of the digested extracts, but without a good correspondence with the levels of polyphenols found by HPLC. Hidalgo et al. [22] concluded that this increase was not justified by the possible release of Maillard reaction compounds in biscuits. Palavecino et al. [43] obtained a similar result, finding an increase in antioxidant capacity both after cooking and after *in vitro* digestion of samples of sorghum gluten-free pasta and suggested that this increase was linked to the release of bound phenolics during digestion. Similarly, intestinal digestates of durum or bread wheat presented a higher antioxidant capacity than raw and cooked samples [44].

## 4. Conclusions

Enriching gluten-free pasta with tomato and linseed industrial by-products is a good alternative to improve the nutritional composition of foods. In cooked pasta, their addition significantly increased the content of bioactive compounds compared to the control. Even after *in vitro* digestion, the pasta with tomato and linseed waste retained a relevant nutritional advantage over the control, indicating that the addition of these by-products is a viable option to improve the nutritional quality of gluten-free pasta. Bioaccessibility was the highest for the free phenolic compounds, whereas carotenoids and tocols were only partially available for absorption. Further research is needed to evaluate technological quality of these products and their acceptability by consumers.

## Figures and Tables

**Table 1:** Composition of carotenoids (mean ± standard deviation; mg/kg DM) in cooked pasta and *in vitro* digested pasta prepared with rice and fava bean flour (control) and enriched with different percentages of tomato waste (TO) or linseed (LI).

	Control	10% TO	15% TO	10% LI	15% LI
**Cooked**					
Lycopene + β-carotene	0.08 ^d^ ± 0.00	0.60 ^b^ ± 0.01	0.76 ^a^ ± 0.01	0.07 ^d^ ± 0.01	0.10 ^c^ ± 0.00
β-cryptoxanthin	0.04 ± 0.00	0.05 ± 0.00	0.05 ± 0.00	0.05 ± 0.01	0.06 ± 0.01
Lutein	0.88 ^b^ ± 0.03	0.65 ^c^ ± 0.01	0.65 ^c^ ± 0.05	0.91 ^b^ ± 0.09	1.13 ^a^ ± 0.09
Zeaxanthin	0.03 ^c^ ± 0.00	0.06 ^ab^ ± 0.00	0.07 ^a^ ± 0.01	0.05 ^b^ ± 0.00	0.07 ^ab^ ± 0.01
**Digested**					
*Soluble*					
Lycopene + β-carotene	0.06 ^b^ ± 0.03	0.30 ^a^ ± 0.02	0.35 ^a^ ± 0.01	0.05 ^b^ ± 0.04	0.08 ^b^ ± 0.01
β-cryptoxanthin	0.02 ± 0.00	0.02 ± 0.00	0.02 ± 0.00	nd	nd
Lutein	0.58 ^a^ ± 0.03	0.28 ^c^ ± 0.01	0.26 ^c^ ± 0.01	0.58 ^a^ ± 0.03	0.53 ^b^ ± 0.02
Zeaxanthin	0.04 ± 0.01	0.02 ± 0.00	0.01 ± 0.01	0.02 ± 0.01	0.03 ± 0.00
*Insoluble*					
Lycopene + β-carotene	0.03 ^c^ ± 0.01	0.23 ^b^ ± 0.03	0.36 ^a^ ± 0.04	0.02 ^c^ ± 0.01	0.02 ^c^ ± 0.01
β-cryptoxanthin	0.02 ± 0.01	0.01 ± 0.01	0.02 ± 0.01	0.01 ± 0.00	0.01 ± 0.00
Lutein	0.26 ± 0.04	0.33 ± 0.01	0.28 ± 0.02	0.26 ± 0.02	0.31 ± 0.00
Zeaxanthin	0.04 ± 0.01	0.02 ± 0.01	0.02 ± 0.01	0.01 ± 0.00	0.01 ± 0.01

nd: not detected, i.e., lower than the detection limit. Different letters indicate significant difference (*p* < 0.05) among samples in the row.

**Table 2:** Composition of tocols (mean ± standard deviation; mg/kg DM) in cooked pasta and *in vitro* digested pasta prepared with rice and fava bean flour (control) and enriched with different percentages of tomato waste (TO) or linseed (LI).

	Control	10% TO	15% TO	10% LI	15% LI
**Cooked**					
α-tocopherol	nd	1.08 ± 0.09	1.08 ± 0.01	nd	nd
β-tocotrienol	nd	nd	nd	5.05 ^b^ ± 0.80	9.30 ^a^ ± 0.69
γ-tocopherol	6.35 ^d^ ± 0.06	7.24 ^c^ ± 0.29	11.27 ^a^ ± 0.20	9.01 ^b^ ± 0.04	11.73 ^a^ ± 0.63
γ-tocotrienol	0.66 ^b^ ± 0.02	0.51 ^c^ ± 0.02	0.46 ^c^ ± 0.02	0.81 ^a^ ± 0.02	0.68 ^b^ ± 0.05
**Digested**					
*Soluble*					
α-tocopherol	nd	0.27 ^b^ ± 0.05	0.31 ^b^ ± 0.03	0.38 ^a^ ± 0.02	0.34 ^ab^ ± 0.02
β-tocopherol	nd	0.20 ± 0.08	0.12 ± 0.03	0.16 ± 0.02	0.30 ± 0.03
β-tocotrienol	nd	nd	nd	3.92 ± 0.52	6.09 ± 0.83
γ-tocopherol	1.83 ^d^ ± 0.02	3.72 ^c^ ± 0.24	4.20 ^c^ ± 0.25	6.03 ^b^ ± 0.24	7.48 ^a^ ± 0.49
γ-tocotrienol	0.15 ^c^ ± 0.02	0.28 ^b^ ± 0.02	0.27 ^b^ ± 0.00	0.31 ^ab^ ± 0.03	0.39 ^a^ ± 0.05
δ-tocopherol	0.15 ± 0.01	nd	nd	nd	nd
*Insoluble*					
α-tocopherol	0.17 ^c^ ± 0.02	0.75 ^b^ ± 0.04	0.92 ^a^ ± 0.04	0.06 ^d^ ± 0.01	0.07 ^d^ ± 0.01
β-tocotrienol	nd	nd	nd	1.82 ± 0.59	3.68 ± 0.79
β-tocopherol	0.19 ± 0.01	0.10 ± 0.03	0.17 ± 0.03	0.16 ± 0.00	nd
γ-tocopherol	3.24 ^b^ ± 0.05	3.40 ^b^ ± 0.08	5.03 ^a^ ± 0.08	1.94 ^c^ ± 0.76	2.58 ^bc^ ± 0.54
γ-tocotrienol	0.27 ^a^ ± 0.01	0.17 ^bc^ ± 0.02	0.21 ^bc^ ± 0.03	0.20 ^ab^ ± 0.02	0.14 ^c^ ± 0.05

nd: not detected, i.e., lower than the detection limit. Different letters indicate significant difference (*p* < 0.05) among samples in the row.

**Table 3:** Composition of free polyphenols and tryptophan (mean ± standard deviation; mg/kg DM) in cooked pasta and *in vitro* digested pasta prepared with rice and fava bean flour (control) and enriched with different percentages of tomato waste (TO) or linseed (LI).

	Control	10% TO	15% TO	10% LI	15% LI
**Cooked**					
*Phenolic acids*					
Protocatechuic	48.1 ± 2.1	54.8 ± 2.5	52.3 ± 0.7	52.3 ± 2.6	52.7 ± 4.8
*p*-hydroxybenzoic	1.96 ± 0.08	nd	nd	nd	nd
Syringic	8.80 ^c^ ± 0.29	12.50 ^b^ ± 1.46	21.39 ^a^ ± 1.96	10.95 ^bc^ ± 0.22	9.97 ^bc^ ± 0.09
*p*-coumaric	5.45 ^a^ ± 0.02	2.59 ^b^ ± 0.59	2.33 ^bc^ ± 0.30	1.69 ^cd^ ± 0.07	1.49 ^d^ ± 0.02
Ferulic	3.58 ^d^ ± 0.54	4.03 ^cd^ ± 0.09	4.87 ^b^ ± 0.42	4.80 ^bc^ ± 0.12	5.71 ^a^ ± 0.15
*Flavonoids*					
Catechin	129.5 ± 11.2	139.9 ± 3.7	139.4 ± 4.4	147.8 ± 9.2	142.5 ± 0.8
Epicatechin	2.48 ^c^ ± 0.42	2.55 ^c^ ± 0.05	2.38 ^c^ ± 0.08	3.40 ^b^ ± 0.02	4.32 ^a^ ± 0.15
Rutin	nd	4.54 ^b^ ± 0.23	8.23 ^a^ ± 0.67	nd	nd
Quercetin	nd	2.35 ± 0.17	3.36 ± 0.41	nd	nd
Quercetin der	0.86 ^c^ ± 0.03	1.07 ^bc^ ± 0.06	1.47 ^a^ ± 0.21	1.32 ^ab^ ± 0.09	1.04 ^bc^ ± 0.16
Naringenin	nd	13.3 ^b^ ± 0.1	22.7 ^a^ ± 0.7	nd	nd
Apigenin	0.23 ± 0.01	0.20 ± 0.03	0.22 ± 0.04	0.36 ± 0.18	0.36 ± 0.20
Tryptophan *	4.76 ^d^ ± 0.43	14.09 ^c^ ± 0.57	21.48 ^c^ ± 0.51	15.36 ^b^ ± 0.57	19.74 ^a^ ± 0.38
**Digested**					
*Phenolic acids*					
Protocatechuic	48.9 ^a^ ± 0.8	49.9 ^a^ ± 0.4	44.5 ^b^ ± 1.8	47.7 ^ab^ ± 2.8	44.5 ^b^ ± 0.6
Syringic	19.4 ^b^ ± 0.9	27.0 ^a^ ± 2.6	28.8 ^a^ ± 0.7	19.6 ^b^ ± 0.4	20.2 ^b^ ± 0.9
*p*-coumaric	2.71 ^bc^ ± 0.18	2.82 ^b^ ± 0.93	4.27 ^a^ ± 0.03	1.79 ^cd^ ± 0.07	1.58 ^d^ ± 0.02
Ferulic	4.40 ^c^ ± 0.62	6.14 ^b^ ± 0.54	8.58 ^a^ ± 0.03	5.09 ^d^ ± 0.13	6.05 ^d^ ± 0.16
*Flavonoids*					
Catechin	127.9 ± 1.1	121.6 ± 6.7	117.9 ± 6.2	122.2 ± 4.2	116.1 ± 3.1
Epicatechin	19.3 ^b^ ± 0.05	17.7 ^b^ ± 2.86	28.1 ^a^ ± 0.05	28.8 ^a^ ± 4.84	31.8 ^a^ ± 0.86
Naringenin	nd	4.82^b^ ± 0.10	6.86^a^ ± 0.13	nd	nd
Tryptophan *	227.8 ^c^ ± 2.6	267.5 ^c^ ± 26.72	267.4 ^c^ ± 3.5	339.7 ^b^ ± 21.6	403.5 ^a^ ± 4.8

nd: not detected, i.e., lower than the detection limit. Different letters indicate significant difference (*p* < 0.05) among samples in the row; * semi-quantitative determination.

**Table 4:** Composition of bound polyphenols (mean ± standard deviation; mg/kg DM) in cooked pasta and *in vitro* digested pasta prepared with rice and fava bean flour (control) and enriched with different percentages of tomato waste (TO) or linseed (LI).

	Control	10% TO	15% TO	10% LI	15% LI
**Cooked**					
*Phenolic acids*					
Protocatechuic	0.86 ^cd^ ± 0.19	0.62 ^d^ ± 0.09	1.23 ^c^ ± 0.08	21.71 ^b^ ± 0.09	37.15 ^a^ ± 0.21
*p*-hydroxybenzoic	0.97 ^d^ ± 0.01	2.55 ^b^ ± 0.30	3.47 ^a^ ± 0.04	1.98 ^c^ ± 0.03	3.46 ^a^ ± 0.11
Caffeic	nd	0.46 ^c^ ± 0.07	2.08 ^ab^ ± 0.18	1.29 ^bc^ ± 0.06	3.11 ^a^ ± 1.33
*p*-coumaric	3.57 ᵉ ± 0.32	17.21 ^b^ ± 2.21	29.98 ^a^ ± 0.69	6.58 ^d^ ± 0.53	8.81 ^c^ ± 0.32
Sinapic	0.25 ^c^ ± 0.02	0.61 ^c^ ± 0.11	0.70 ^c^ ± 0.03	2.30 ^b^ ± 0.22	3.96 ^a^ ± 0.41
Ferulic	94.7 ^b^ ± 1.2	62.7 ^c^ ± 4.0	64.5 ^c^ ± 0.2	95.9 ^b^ ± 0.1	119.8 ^a^ ± 1.5
Cinnamic der	0.89 ^c^ ± 0.01	4.44 ^b^ ± 0.56	6.87 ^a^ ± 0.02	0.87 ^c^ ± 0.07	0.83 ^c^ ± 0.16
*Flavonoids*					
Quercetin	1.00 ^c^ ± 0.01	28.0 ^b^ ± 0.4	35.4 ^a^ ± 3.4	1.42 ^c^ ± 0.19	0.89 ^c^ ± 0.02
Naringenin	0.54 ^c^ ± 0.01	20.6 ^b^ ± 0.2	34.0 ^a^ ± 0.1	1.90 ^c^ ± 0.14	0.40 ^c^ ± 0.02
**Digested**					
*Phenolic acids*					
Protocatechuic	nd	nd	nd	23.71 ^b^ ± 2.11	39.26 ^a^ ± 3.32
*p*-hydroxybenzoic	2.41 ^cd^ ± 0.47	7.31 ^b^ ± 0.69	8.64 ^a^ ± 0.55	2.16 ^d^ ± 0.27	3.55 ^c^ ± 0.00
Caffeic	nd	2.90 ^b^ ± 0.28	3.37 ^ab^ ± 0.04	2.91 ^b^ ± 0.10	3.67 ^a^ ± 0.28
*p*-coumaric	2.96 ^d^ ± 0.15	14.40 ^b^ ± 1.95	19.81 ^a^ ± 1.28	6.40 ^c^ ± 0.63	7.39 ^c^ ± 0.82
Sinapic	0.68 ^c^ ± 0.14	0.82 ^c^ ± 0.18	0.87 ^c^ ± 0.03	2.08 ^b^ ± 0.15	3.27 ^a^ ± 0.12
Ferulic	79.4 ^b^ ± 11.0	63.1 ^c^ ± 4.1	53.3 ^c^ ± 1.6	93.7 ^ab^ ± 6.5	102.5 ^a^ ± 1.1
Cinnamic der	0.85 ^bc^ ± 0.02	0.97 ^a^ ± 0.04	0.83 ^c^ ± 0.03	0.93 ^ab^ ± 0.00	0.99 ^a^ ± 0.06
*Flavonoids*					
Epicatechin	0.20 ^b^ ± 0.09	1.21 ^a^ ± 0.30	0.59 ^b^ ± 0.01	nd	0.15 ^b^ ± 0.00
Quercetin	nd	10.0 ^b^ ± 0.6	12.7 ^a^ ± 0.2	nd	nd
Naringenin der	3.07 ± 0.28	2.76 ± 0.12	2.81 ± 0.38	2.87 ± 0.48	3.76 ± 0.01
Naringenin	nd	36.2 ^b^ ± 1.7	54.2 ^a^ ± 3.3	nd	nd

nd: not detected, i.e., lower than the detection limit. Different letters indicate significant difference (*p* < 0.05) among samples in the row.

## Data Availability

The original contributions presented in this study are included in the article/Supplementary Materials. Further inquiries can be directed to the corresponding author.

## Supplementary Materials

The following supporting information can be downloaded at: https://www.mdpi.com/article/10.3390/foods13223700/s1. Figure S1: Control cooked and raw gluten-free pasta samples and gluten-free pastas enriched with 10% or 15% of tomato waste (TO) or linseed cake (LI).
