# Supplementary material for: Antioxidant Bioaccessibility of Cooked Gluten-Free Pasta Enriched with Tomato Pomace or Linseed Meal
Source: Foods. 2024 Nov 20;13(22):3700. doi: 10.3390/foods13223700 (PMC11593769; doi:10.3390/foods13223700)
Supplement: Supplementary file 1 [file foods-13-03700-s001.zip › foods-3313430-supplementary.pdf]

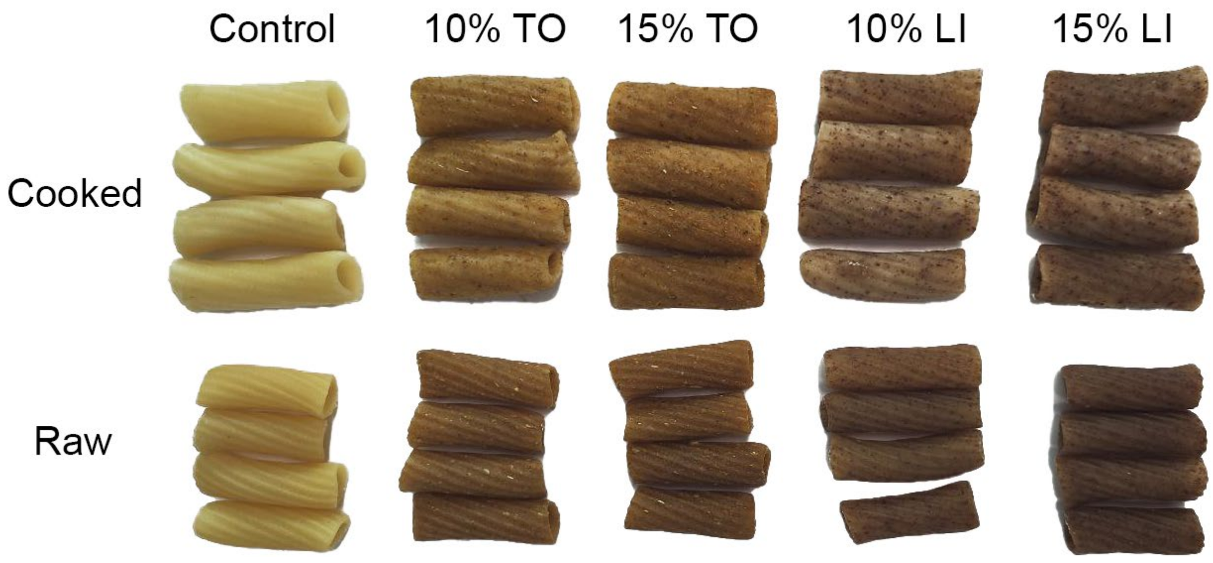

Figure S1. Control cooked and raw gluten-free pasta samples and gluten-free pastas enriched with 10% or 15% of tomato waste (TO) or linseed cake (LI).
